# Understanding Unemployment Normalization: Individual Differences in an Alternative Experience With Unemployment

**DOI:** 10.3389/fpsyg.2020.525506

**Published:** 2020-12-22

**Authors:** Claude Houssemand, Steve Thill, Anne Pignault

**Affiliations:** ^1^Department of Education and Social Work, Institute for Lifelong Learning and Guidance, University of Luxembourg, Esch-sur-Alzette, Luxembourg; ^2^Psychology & Neuroscience Laboratory (2LPN), University of Lorraine, Nancy, France

**Keywords:** unemployment, normalization, individual differences, personality, coping, perceived health, subjective well-being

## Abstract

Unemployment is a major concern of societies and people around the world. In addressing this phenomenon, the literature has suggested a change in unemployed people’s perceptions of this transition period. In this paper, we apply a differential approach to explore the concept of unemployment normalization, an individual emotional regulation process. The results show how the global socioeconomic context and some individual and psychological variables influence the normalization of unemployment. Thus, the age of the person but also work involvement, coping strategies, locus of control, and level of self-esteem have indirect differential effects, mediated by unemployment normalization dimensions, on unemployed people’s perceived health. Only neuroticism has a direct link to subjective well-being. These results offer a new understanding of the perception of unemployment and are also discussed in the area of career and vocational counseling.

## Introduction

Unemployment is a rather important negative life event (ranking 13th out of 51 events) with a slightly higher impact for men (Hobson et al., 1998). Thus, it has to be considered a very high life stressor. Nevertheless, different individuals respond to unemployment in different ways, and the differential mechanisms and processes used to deal with unemployment need to be studied more (Gowan, 2014). The consequences of unemployment on subjective well-being (SWB), health, identity, and distress have already been widely studied; scholars agree about the deleterious effects for the unemployed (McKee-Ryan et al., 2005; Wanberg, 2012). Nevertheless, a recent body of research has shown that complex mechanisms of emotional regulation during unemployment may explain inter- and intraindividual variability in experiences and feelings about this professional transition period (Houssemand and Pignault, 2017; Pignault and Houssemand, 2017; Thill et al., 2018). Thus, by losing revenue but also by being deprived of the well-known beneficial latent functions of work (Jahoda, 1997; Creed and Evans, 2002; Paul and Batinic, 2010), individuals must somehow adapt to or cope with this stressful situation through psychological compensation (Lazarus and Folkman, 1984; Latack and Havlovic, 1992). In unemployment research, some authors have suggested that a coping strategy that can effectively deal with unemployment may compensate for its negative effects on mental health (Fryer and Payne, 1984; Starrin and Larsson, 1987; Leana et al., 1998; Patton and Donohue, 1998; Lin and Leung, 2010). Kinicki and Latack (1990) identified two strategies for avoiding the impact of unemployment: “distancing from loss” and “job devaluation.” They then paved the way for a body of research that focuses on the processes of intraindividual regulation of job loss and unemployment. This regulation is considered to be linked to individual psychological dimensions (Latack et al., 1995) but also depends on regional economic factors and social norms related to work and unemployment (Houssemand and Pignault, 2017; Pignault and Houssemand, 2018). These recent results indicate that the history of unemployment in a country or region has effects on a person’s feelings and experiences, a finding that appears to contradict Paul and Moser’s (2009) robust and well-known results. Even though the regional unemployment rate alone is not a good socioeconomic indicator of unemployment, it now seems clear that different relationships can exist between occupational status and SWB (e.g., Stam et al., 2015; Buffel et al., 2017). Thus, whereas the negative effects of unemployment are felt in the same way by unemployed people from culturally and geographically close countries, other psychological dimensions have differential effects on this subjective experience (Hahn et al., 2015; Houssemand and Pignault, 2017; Pignault and Houssemand, 2017). In sum, there seems to be an individual cognitive mechanism for regulating unemployment, but it depends on the contexts in which unemployed people live.

In this vein and drawing on Ashforth and Kreiner’s (2002) work, which showed how people *normalize* certain “extraordinary” situations in an organizational context in order to make them seem more acceptable and more ordinary, Pignault and Houssemand (2018) suggested the concept of *unemployment normalization*. Because the unemployment situation is stressful, the authors described a process by which unemployment is normalized, consisting of a form of emotional regulation involving a process of cognitive reappraisal (Gross and Thompson, 2007). Nevertheless, without going so far as to identify the normalization of unemployment as a social construct, it is important to understand that this concept depends on the social, cultural, and economic circumstances in which unemployed people try to regulate their emotions. Thus, the normalization of unemployment must be understood as a multidimensional adaptive and cognitive response to a situation that is considered new and stressful (Pignault and Houssemand, 2018). In this sense, normalizing unemployment would enable a person to implement a self-regulation strategy (certainly unconscious) to maintain their SWB in a positive way or at least in a way that is as high as possible under these conditions (Rasmussen et al., 2006; Wanberg, 2012) and to confer resilience (Johnson et al., 2016).

### Coping With Unemployment

Coping has been studied a great deal in the field of work stress (for an extensive review, see Dewe et al., 2010). As in all stressful situations, people use cognitive appraisals that are composed of primary appraisals (the process by which the situation is analyzed) and secondary appraisals (the process of choosing coping mechanisms that will determine the impact of the stressor on well-being). Depending on the nature of the environment in which the stress occurs, coping strategies might change, and different response behaviors might work better or worse for certain stressors. Nevertheless, people have coping styles that represent the general habits they apply to respond to stressors. Coping strategies have been widely studied (for a review, see Folkman and Moskowitz, 2004), and there are many models of coping strategies. For example, the first one empirically distinguished problem-focused coping from emotion-focused coping (Lazarus and Folkman, 1984), whereas others have provided systematic reviews of different coping measurement tools describing hundreds of coping behaviors (e.g., Skinner et al., 2003). Some overlap exists between models, and the more complex models can often be summarized with simpler ones. Nevertheless, stressors are generally identified as specific to different contexts, and environmental situations can explain the coping responses that are used. The specific context of a lack of job security, including actual unemployment, has an important effect on subjective strain and well-being (Probst, 2009). A coping strategy based on proactive behaviors seems to stabilize or increase the well-being of unemployed people and reduce their uncertainty about employment (Mantler et al., 2005).

Some reviews have been conducted on coping with unemployment (e.g., Waters, 2000; Gowan, 2014), but such reviews have concluded that there is a need for more research, especially analyses of the process of coping with unemployment (e.g., Kinicki et al., 2000; Waters, 2000; McKee-Ryan et al., 2005; Rudisill et al., 2010). These models may be helpful for improving our understanding of the coping process. We can summarize this field of research by listing some important steps.

On the basis of Lazarus and Folkman’s (1984) research on differential coping and appraisal processes during stressful events, DeFrank and Ivancevich (1986) proposed one of the first models related to coping with unemployment. Based on organizational (e.g., company history or financial condition) and individual (e.g., age, education, or chronic health) risk factors, a worker can lose his or her job, which immediately impacts the person’s income (e.g., money) and social status. Some personal (e.g., personality or flexibility), social (e.g., social support or impact on family), economic (e.g., climate or location), and job-related variables (e.g., involvement or satisfaction) moderate the differential perception or appraisal of a layoff, coping attempts, and effects (e.g., physical or psychological).

Leana and Feldman (1988) model of job loss was designed to explain individuals’ reactions to this situation. Because it is a stressful event, job loss implies physiological changes but also cognitive appraisal and emotional arousal, which drive how people cope with unemployment. Coping strategies are moderated by personality (e.g., locus of control or self-esteem) and situational (e.g., labor economic conditions or social support) factors. These coping processes may affect the job attainment which influence some outcomes (e.g., job attitudes or general health).

An extension of the two previous models was presented by Latack et al. (1995). This model, which was based on coping theory, control theory, and self-efficacy, tries to explain the coping process used to maintain psychological equilibrium during unemployment. It involves cybernetic control process (Edwards, 1992) because job loss is considered a stressful situation where individuals compare their actual situation to economic, psychological, physiological, and social standards. People’s appraisals of this discrepancy impact their coping goals. Finally, coping strategies are determined by coping goals moderated by coping resources and coping efficacy.

Gowan and Gatewood’s (1997) model subdivides the process of coping with involuntary job loss into four steps. The first consists of individual and situational coping resources that are causal antecedents of people’s reactions to stress. The second one is represented by cognitive appraisal (e.g., reversibility or perceived fairness) and coping strategies (problem, symptom, or emotion-focused coping) as mediating processes. At the third level, coping strategies influence immediate effects of job loss, which are psychological affects (especially distress) and reemployment status. Finally, the final level of the model includes the long-term effects or outcomes (e.g., psychological, social, and physiological well-being).

Waters (2000) criticized previous models of the process of coping with unemployment because it proposed that coping is a stable disposition of a person and, thus, it failed to completely explain the coping process. The main objections against the trait-based approach to studying coping processes are (a) a failure to consider permanent and constant changes in coping and its un-static reality (Lazarus and Folkman, 1984), (b) the consideration of coping as a unidirectional phenomenon even if the relationship between the environment and coping is certainly bidirectional (e.g., Moore and Cooper, 1998), (c) the confounding of the impact of coping efforts and coping outcomes, and finally (d) no direct examination of cognitive appraisal during unemployment. The new model proposed by Waters (2000) considered “non-recursive relationships between stressors, cognitive appraisals, coping efforts and psychological health during unemployment” (p. 169).

Recently, a new model of the process of coping with unemployment was proposed by Pignault and Houssemand (2018). Based on the previous model, an intermediate process was integrated into this new model as a moderator between the individual (e.g., locus of control or coping strategies), social (e.g., norms or values), and economic (e.g., unemployment history or employment rate) characteristics of unemployment and outcomes (e.g., stress or well-being). This normalization process is an emotional regulation process based on cognitive reappraisal (reappraisal that views unemployment as a normal and inevitable phase in a person’s career path and as the result of external circumstances). The outcome of this process is that a person’s feelings about being unemployed are less negative, and stress may decrease.

**FIGURE 1:**
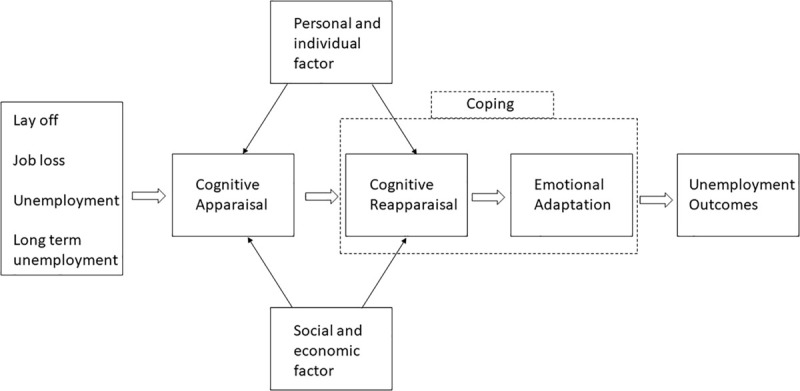
Process of coping with unemployment – a literature summary proposal.

Based on these previous studies and models, it is possible to summarize the potential process of coping with unemployment (broadly interpreted) in Figure 1.

### Unemployment Normalization

Unemployment normalization depends on four interrelated individual emotional or cognitive dimensions (Pignault and Houssemand, 2017). The authors named these four dimensions *negative perceptions of unemployment*, *positive perceptions of unemployment*, *unemployment justifications*, and the *unemployment norm*.

On an emotional level, individuals experience negative and positive feelings about their unemployment status, represented by the negative and positive perception dimensions, respectively. These two contradictory feelings, which are moderately correlated with each other, seem to indicate opposing but simultaneous (not sequential) feelings about being unemployed (alternating periods of negative and positive feelings). Thus, this situation can have harmful effects on unemployed people but simultaneously provide them with positive outcomes (more time for personal activities or an opportunity to reflect on possible career changes).

On a cognitive level, two dimensions that are moderately correlated with each other are based on the individual explanations given for being unemployed. The first concerns the external justifications given (e.g., companies, the economic crisis), represented by the unemployment justifications dimension. The second is a kind of fatalistic explanation of the unemployment situation that mirrors social and economic changes in modern societies (unemployment is unavoidable in today’s career paths), which is the unemployment norm.

The authors found positive correlations between the negative perceptions of unemployment and its justifications and between the positive perceptions of unemployment and the unemployment norm. Finally, they found that negative perceptions of unemployment had a strong negative impact on mental health, whereas positive perceptions preserved psychological well-being.

**FIGURE 2:**
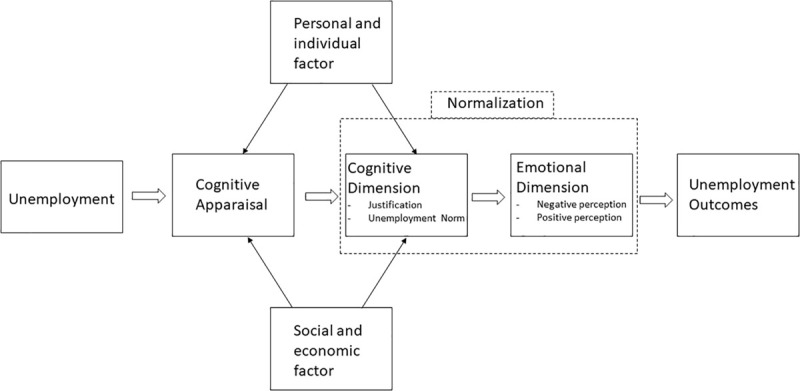
Consideration of normalization as a coping strategy.

The extant scholarship has explored a variety of coping strategies among the unemployed but has not yet considered strategies as shaped by the new normalization construct. A consideration of normalization as a coping strategy is summarized in Figure 2.

### The Differential Approach to the Experience of Unemployment

Other studies have already highlighted the importance of effects of socioeconomic and cultural dimensions on the normalization of unemployment (Houssemand and Pignault, 2017; Pignault and Houssemand, 2017). Unemployed people living in a more favorable socioeconomic context and in a region with less history of unemployment tend to identify fewer positive aspects of unemployment, consider this period less normal in their careers, and less often justify their situation by naming external factors. Nevertheless, beyond these initial findings, individual differences within the same regional context exist, and such differences suggest the existence of personal characteristics that influence the use of regulation strategies. The individual determinants of the unemployment experience have been studied previously, but any empirical confirmation of whether links exist between people’s psychological characteristics and unemployment normalization have yet to be presented.

Work centrality (McKee-Ryan et al., 2005; Wanberg, 2012) and work commitment (Paul and Moser, 2006) are psychological constructs that are often taken into account to better understand the effects and experiences of unemployed people. Studies have shown that the people most engaged in their work are also those who have a more negative experience with being unemployed. Such people find themselves in a situation of cognitive dissonance when deprived of an essential and organizing principle in their lives. Thus, work centrality may have an effect on the unemployment normalization and increase people’s negative feelings about it.

In addition, perceived control is another psychological construct that is traditionally considered in related studies. Nevertheless, perceived control is usually not studied as the only psychological dimension related to unemployment but in relation to coping with unemployment (Wanberg, 1997), job search strategies (Kanfer et al., 2001), and reemployment (Ginexi et al., 2000). In this vein, Petrosky and Birkimer (1991) found a negative relationship between internal locus of control and emotion-focused coping strategies. Moreover, some studies have shown that the effect of unemployment on SWB can be compensated for when people can draw on certain types of coping strategies to deal with the situation (Fryer and Payne, 1984; Starrin and Larsson, 1987; Leana et al., 1998; Patton and Donohue, 1998; Lin and Leung, 2010). Thus, they found that the negative impact of unemployment was greater for people using an emotion-focused coping strategy. We therefore expected that locus of control and coping variables would influence unemployed individuals’ unemployment normalization.

Many studies have further highlighted the influence of personality variables and self-esteem on the experience of unemployment and return to work. In terms of personality, Creed and Evans (2002) showed that the manifest and latent benefits of work were significantly associated with psychological well-being but qualified these results by showing that the variance in terms of SWB is explained primarily by personality dimensions and particularly by neuroticism. Unemployment is probably perceived less negatively by people with a low level of neuroticism even if this link may be discussed (Kokkonen and Pulkkinen, 2001; Boyce et al., 2015). Self-esteem has also been shown to play a moderating role by reducing the level of psychological distress and increasing SWB and the motivation to seek employment (Rowley and Feather, 1987; Wanberg et al., 2005). An unemployed person who maintains good self-esteem will perceive the unemployment situation as less out of their control, as less stressful, and thus as more normalized.

Finally, some other variables that significantly influence the unemployment experience and the SWB of the unemployed should be included as determinants of the level of unemployment normalization. For instance, jobseekers’ age and gender have led to differential effects in the relationships with a person’s unemployment experience, and such differences have been found to be a function of perceived norms, work centrality, and career commitment (Broomhall and Winefield, 1990; Paul and Moser, 2009; Strandh et al., 2013). Moreover, the duration of unemployment and the recurrence of unemployment have been linked to how unemployment is experienced (Hahn et al., 2015). Researchers do not fully agree on the effect of unemployment duration on the experience of the situation. Nevertheless, studies have shown that timing plays an important role in the process of whether people adapt or not, depending on the duration of their unemployment (Clark, 2006; Paul and Moser, 2009; Wanberg et al., 2012a,b). Concerning the recurrence of periods of unemployment, in a longitudinal study, Luhmann and Eid (2009) found that life satisfaction decreased with repeated unemployment, and Booker and Sacker (2012) wondered if unemployment recurrence led to adaptation or sensitization. Thus, these individual non-psychological variables can influence the way of normalizing unemployment, influence one’s experience of this professional transition, and therefore potentially affect the mental health of the unemployed.

### The Present Study

Considering that normalization is a regulating mechanism for unemployment, it becomes important to rethink the relationships between unemployed people’s individual characteristics and their mental health. Indeed, it is probable that the opportunity of emotionally regulating the state of being unemployed depends on a set of individual psychological dimensions (personality, locus of control, coping strategies, and work centrality) and non-psychological characteristics (age, sex, and unemployment history). The dimensions of the unemployment normalization also depend on the socioeconomic characteristics of the unemployed person’s living context. Finally, the unemployment normalization should have a differential impact on jobseekers’ mental health. Thus, as proposed by a heuristic model of unemployment normalization (Pignault and Houssemand, 2018), this emotional regulation of unemployment depends on both psychological and demographic personal characteristics and specific social and economic conditions.

The purpose of this article is to test the heuristic model of the unemployment normalization (Pignault and Houssemand, 2018). As already mentioned, some studies have already highlighted the impact of socioeconomic conditions on this mode of emotionally regulating unemployment (Houssemand and Pignault, 2017; Pignault and Houssemand, 2017). Other research has attempted to understand how the dimensions of the normalization of unemployment interact and compensate for each other, in order to maximally preserve the jobseekers’ SWB (Thill et al., 2018). But, research has yet to take into account unemployed people’s individual characteristics in order to better understand the psychological and individual determinants of the unemployment normalization and its influence on the mental health of the unemployed. This study attempts to address this gap and provide several main hypotheses that are based on previous studies.

• H1: Work centrality is linked to the experience of unemployment. It is positively correlated with the negative perception of unemployment (people for whom work is important in life live in bad times) and, conversely, it is negatively correlated with a positive perception of unemployment. It is also negatively linked to well-being.
• H2: Internal locus and emotion-focused coping are linked to the emotional dimension of unemployment normalization and to well-being. People with a more prominent internal causal attribution do not feel the negative effects of unemployment as profoundly and have better health. People with emotion-focused coping are more affected by unemployment, feel its negative effects more profoundly, and have lower well-being.
• H3: Unemployment is perceived less negatively by people with a low level of neuroticism.
• H4: A high level of self-esteem decreases stress and increases well-being. Self-esteem is negatively linked to a negative perception of unemployment and positively linked to well-being.

## Materials and Methods

### Participants

The sample consisted of 1,038 French-speaking unemployed people (defined as people above a specific age who are currently available for work, seeking work, but without work during some reference period, International Labour Organisation, 2000), of whom 611 were in Luxembourg and 427 in France, contacted during their mandatory individual appointments with state employment agencies. Participants were 38.53 years old on average (*SD* = 11.31); 51.6% were women; 54.5% of them had been unemployed at least once previously; 63.8% received unemployment benefits; and the majority of them (44.7%) had been unemployed for less than 6 months this time around (20.3%: 6 months to 1 year, 24.3%: 1–3 years, 10.7%: more than 3 years). They participated voluntarily at state career centers in Lorraine (*Pôle Emploi*) and Luxembourg (*Agence pour le développement de l’emploi*: *Adem*). Pôle Emploi and Adem are national public employment agencies that were partners of the present study, which was a part of a broader research program funded by the *National Research Fund of Luxembourg* (CORE Program: Project UnemployNorm, under grant number C13/SC/5885577). The state employment agencies gave access to their buildings, announced the study to unemployed people (by email, the press and during follow-up meetings with their guidance professionals), and introduced the researchers to job seekers. This assistance by the agencies helped to achieve a high response rate by unemployed people, as their involvement increased the trust felt by potential respondents. In this sense, the sample was made up of people representing the vast majority of jobseekers officially registered with the public employment services, and thus featuring in the official unemployment figures in France and Luxembourg.

Anonymity and confidentiality were guaranteed. Information concerning the study’s goals, the researchers’ identities, and data processing was provided to participants orally and in writing. The two data collection sites are geographically close but have very different socioeconomic contexts. Thus, in this region of France (Lorraine), the unemployment rate is very high (above 10%), whereas it is rather low in Luxembourg (6%, with the latter being one of the lowest in the European Union; Eurostat, 2018). In addition, the history of unemployment is different between these two countries: There has long been unemployment in France, but it is quite recent in Luxembourg. For example, in 1996, France’s unemployment rate was 11.6%, and Luxembourg’s was 2.9%. Luxembourg is considered a “favorable labor market” (Houssemand and Meyers, 2011, p. 378). Comparisons between these two employment regions can thus further the understanding of the importance of the socioeconomic context on the unemployment experience.

### Measures

A multipart questionnaire was administered to participants.

#### Unemployment Normalization Questionnaire (Pignault and Houssemand, 2017)

Answers were given to 16 items broken down into four dimensions on a 4-point Likert scale ranging from 1 (*strongly disagree*) to 4 (*strongly agree*). The scale is coherent with the model depicting the coping with unemployment processes. It described a cognitive dimension, composed of two factors. In this, unemployment is perceived as a normal stage in professional careers, and so is considered to be a norm, *unemployment norm*, (example: *Unemployment is now an inevitable stage in life*), and through *external justification* of unemployment (example: *Unemployment is a result of the crisis*). This cognitive dimension has impacts on an emotional dimension, described as a *negative perception* of unemployment factor (example: *Since I have been unemployed, I feel different from others*) and a *positive perception* of unemployment factor (example: *Since becoming unemployed, I feel better than before*). The complete scale has already been published (Pignault and Houssemand, 2017).

#### General Health Questionnaire (Goldberg, 1972)

This mental health questionnaire was selected because it has 12 items, good psychometric characteristics (Hankins, 2008), and enables international comparisons because of its temporal and cross-cultural invariance (e.g., Mäkikangas et al., 2006). A high score on this scale indicates more severe mental health problems, whereas a low score reveals good mental health. The respondents have had to judge if different dimensions of their current life were actually changed (example: *Have you recently been able to concentrate on what you are doing?* less than usual, no more than usual, rather more than usual, or much more than usual, with these answers coded, respectively with 0-1-2-3).

#### Rosenberg Self-Esteem Scale (Rosenberg, 1965)

Responses were given to 10 items on a scale ranging from 0 (*strongly disagree*) to 3 (*strongly agree*) (example*: I feel that I have a number of good qualities*). The scale therefore varies from 0 to 30, with a high score indicating a higher level of self-esteem.

#### Control of Unemployment Scale (Houssemand et al., 2019)

The *Multidimensional Health Locus of Control* (MHLC) Scale (Wallston et al., 1978) was used, but the context of the items was changed. The scales were tailored specifically to unemployed people and the situation of being unemployed (Meyers and Houssemand, 2010; Houssemand et al., 2019). Based on Levenson’s (1973) theory, this 16-item scale measures three dimensions of control in situations of unemployment and job-seeking. It uses a 4-point Likert scale, ranging from 0 (*absolutely disagree*) to 3 (*absolutely agree*): internal locus of control (example: *If I take care, I can avoid being unemployed again*); powerful others (example: *Being in regular contact with the administration office is the only way for me to find a job*); and chance (example: *Most of the things that affect my job search happen by chance*).

#### Work Involvement Scale (Warr et al., 1979)

This six-item scale measures work centrality and thus the importance given to this activity (example: *Having a job is very important to me*). Responses range from 1 (*very strongly disagree*) to 7 (*very strongly agree*). Thus, higher scores reflect greater importance of work in the respondent’s life.

#### Way of Coping Checklist (Vitaliano et al., 1985)

Coping was measured with 27 items describing three coping strategies: Problem-focused (example: *I made a plan of action and followed it*); Emotion-focused (example: *I hoped a miracle would happen*); and Social-support coping (example: *I talked to someone to find out about the situation*). Participants’ responses were coded 1 (*No*), 2 (*Somewhat no*), 3 (*Somewhat yes*), and 4 (*Yes*).

#### Neuroticism (Costa and McCrae, 1998)

Neuroticism was assessed with the 12-item *NEOFFI* scale (Costa and McCrae, 1998), a short five-factor omnibus test of personality (example: *I am rarely sad or depressed*). Items were rated on a 5-point scale ranging from 0 (*strongly disagree*) to 4 (*strongly agree*).

These scales were chosen for their psychometric qualities and their frequent use in international studies. All these surveys were written in French. A series of demographic questions were also asked: age (in years), unemployment duration (modalities were: less than 6 months, 6 months to 1 year, 1–3 years, and, more than 3 years), recurrence (first period of unemployment or not), and whether or not the jobseeker was receiving unemployment benefits (in France and Luxembourg, under certain conditions related to age and previous work duration, unemployed people may receive financial assistance from the government while they are looking for work).

### Statistical Analysis

In order to respect the level of measurement of the data (Stevens, 1946), the analyses in this study were based on polychoric correlation matrices between the items on each scale (Carroll, 1961; Muthén, 1984). As a result, structural equation modeling (confirmatory factor analyses and path analysis) used the DWLS estimator (diagonally weighted least squares) in the R-package Lavaan.

## Results

### Homogeneity of Scales

Table 1 presents the internal consistencies of each of the dimensions of each scale. We observed that these values were all very high and close to 1, indicating that the items on each scale or subscale are homogeneous, which allowed us to estimate the latent psychological scores.

**TABLE 1:** Cronbach’s alphas for each dimension.

**Scale/dimensions**	**Alpha**
Unemployment Normalization:	
•Negative perceptions of unemployment •Positive perceptions of unemployment •Unemployment justifications •Unemployment norm	0.77 0.77 0.63 0.76
General Health Questionnaire	0.92
Rosenberg Self-Esteem Scale	0.85
Control of Unemployment Scale	
•Internal locus •External locus •Chance locus	0.69 0.65 0.71
Work Involvement Scale	0.83
French version of Way of Coping Checklist	
•Problem-focused coping •Emotion-focused coping •Social-support coping	0.83 0.79 0.76
NEOFFI Neuroticism Scale	0.87

In general, for all scales, the internal consistencies of each dimension were greater than or very close to.70 and corresponded to the values observed in research using these scales. It was therefore possible to calculate a composite score indicating the individual score of each subject on each of the psychological dimensions measured in this study.

### Links Between the Psychological Dimensions and the Unemployment Normalization

In order to understand the relationships that may exist between the various psychological dimensions and the unemployment normalization, we computed correlations. Table 2 presents all links between all study variables. There was a strong positive relationship between the intensity of negative perceptions of unemployment and mental health problems, work centrality, neuroticism, and emotion-focused coping. So, unemployed people who considered work to be an essential part of their life, who also expressed negative feelings easily, and had not coped well in an active fashion, perceived the unemployment situation to be a more negative experience and that their subjective health had worsened. In addition, the intensity of positive perceptions of unemployment was inversely related to mental health problems and work centrality. Thus, unemployed people who saw some positive aspects to a period of unemployment, had better feelings of subjective health. More generally, these were people for whom work is less essential. Finally, the justification and unemployment norm dimensions were only weakly related to the other psychological variables.

**TABLE 2:** Correlations between normalization dimensions and psychological variables.

(1) Negative perceptions													
(2) Positive perceptions	−0.539***												
95% CI	[−0.582; −0.494]												
(3) Unempl. justifications	0.489***	−0.162***											
95% CI	[0.440; 0.534]	[−0.221; −0.101]											
(4) Unempl. norm	−0.077*	0.336***	0.320***										
95% CI	[−0.138; −0.016]	[0.280; 0.389]	[0.264; 0.375]										
(5) Mental health	0.649***	−0.448***	0.270***	−0.115**									
95% CI	[0.612; 0.684]	[−0.496; −0.397]	[0.212; 0.327]	[−0.175; −0.053]									
(6) Self esteem	−0.397***	0.162***	−0.113**	0.080*	−0.495***								
95% CI	[−0.448; −0.344]	[0.101; 0.221]	[−0.173; −0.051]	[0.013; 0.136]	[−0.540; −0.447]								
(7) Internal locus	−0.076*	0.050	−0.090*	0.050	−0.162***	0.198***							
95% CI	[−0.137; −0.013]	[−0.010; 0.115]	[−0.155; −0.031]	[−0.009; 0.115]	[−0.222; −0.102]	[0.138; 0.257]							
(8) External locus	0.170***	0.079*	0.171***	0.172***	0.080*	−0.233***	−0.050						
95% CI	[0.109; 0.230]	[0.017; 0.141]	[0.110; 0.231]	[0.111; 0.231]	[0.016; 0.139]	[−0.290; −0.173]	[−0.112; 0.012]						
(9) Chance locus	0.192***	0.070*	0.231***	0.225***	0.137***	−0.239***	−0.189***	0.901**					
95% CI	[0.131; 0.251]	[0.007; 0.131]	[0.171; 0.289]	[0.165; 0.283]	[0.076; 0.197]	[−0.296; −0.180]	[−0.248; −0.128]	[0.889; 0.912]					
(10) Work involvement	0.368***	−0.524***	0.159***	−0.118**	0.286***	−0.030	0.129***	0.040	0.000				
95% CI	[0.313; 0.420]	[−0.567; −0.478]	[0.099; 0.219]	[−0.178; −0.056]	[0.229; 0.341]	[−0.094; 0.029]	[0.068; 0.189]	[−0.027; 0.097]	[−0.064; 0.059]				
(11) Problem coping	0.000	−0.010	−0.030	−0.010	−0.113**	0.226***	0.255***	0.020	−0.050	0.150***			
95% CI	[−0.066; 0.059]	[−0.072; 0.052]	[−0.095; 0.029]	[−0.074; 0.051]	[−0.174; −0.052]	[0.166; 0.284]	[0.196; 0.312]	[−0.044; 0.080]	[−0.116; 0.008]	[0.088; 0.209]			
(12) Emotion coping	0.547***	−0.273***	0.215***	−0.030	0.483***	−0.385***	−0.040	0.297**	0.302***	0.237***	0.310***		
95% CI	[0.502; 0.589]	[−0.330; −0.214]	[0.155; 0.274]	[−0.090; 0.034]	[0.434; 0.529]	[−0.436; −0.331]	[−0.106; 0.018]	[0.239; 0.352]	[0.244; 0.357]	[0.178; 0.295]	[0.254; 0.365]		
(13) Social coping	0.229***	−0.103*	0.070*	−0.020	0.143***	−0.010	0.126***	0.173**	0.119**	0.200***	0.785***	0.627***	
95% CI	[0.169; 0.287]	[−0.164; −0.041]	[0.010; 0.134]	[−0.077; 0.048]	[0.082; 0.203]	[−0.068; 0.056]	[0.064; 0.187]	[0.112; 0.233]	[0.057; 0.180]	[0.139; 0.258]	[0.760; 0.807]	[0.588; 0.663]	
(14) Neuroticism	0.544***	−0.216***	0.245**	0.010	0.641***	−0.652**	−0.136***	0.262**	0.316***	0.113**	−0.112**	0.543***	0.159**
95% CI	[0.499; 0.586]	[−0.274; −0.156]	[0.186; 0.302]	[−0.048; 0.075]	[0.603; 0.676]	[−0.686; −0.615]	[−0.196; −0.074]	[0.204; 0.319]	[0.259; 0.371]	[0.052; 0.173]	[−0.173; −0.051]	[0.498; 0.585]	[0.098; 0.218]
	1	2	3	4	5	6	7	8	9	10	11	12	13
													

*p < 0.05, **p < 0.01, ***p < 0.001.

### Links Between Demographic Data and the Unemployment Normalization

In order to better understand the unemployment normalization and the individual differences in its implementation during unemployment, we computed ANOVAs on the four dimensions of normalization and demographic variables. Table 3 presents the results.

**TABLE 3:** ANOVAs on normalization dimensions and demographic variables.

	**Sex *N* = 1005 Men = 48.5% Women = 51.5%**	**Duration *N* = 1005 <6 months = 43.4% 6–12 months = 20.4% 12–36 months = 24.6% >36 months = 10.6%**	**Redundancy *N* = 1004 First period = 45.2% Not first period = 54.8%**	**Unempl. benefits *N* = 1001 Yes = 63.8% No = 36.2%**	**Country *N* = 1009 Luxembourg = 57.9% France = 42.1%**
Negative perceptions	*F*_(__1_,_1003__)_ = 0.08	*F*_(__3_,_1001__)_ = 32.97*** η^2^ = 0.03	*F*_(__1_,_1002__)_ = 0.08	*F*_(__1_,_999__)_ = 0.06	*F*_(__1_,_1007__)_ = 4.03* η^2^ = 0.01
Positive perceptions	*F*_(__1_,_1003__)_ = 0.25	*F*_(__3_,_1001__)_ = 7.16** η^2^ = 0.01	*F*_(__1_,_1002__)_ = 1.73	*F*_(__1_,_999__)_ = 4.20* η^2^ = 0.01	*F*_(__1_,_1007__)_ = 2.84
Justifications	*F*_(__1_,_1003__)_ = 2.64	*F*_(__3_,_1001__)_ = 34.34*** η^2^ = 0.03	*F*_(__1_,_1002__)_ = 11.58*** η^2^ = 0.01	*F*_(__1_,_999__)_ = 1.06	*F*_(__1_,_1007__)_ = 5.04* η^2^ = 0.01
Norm	*F*_(__1_,_1003__)_ = 3.33	*F*_(__3_,_1001__)_ = 1.73	*F*_(__1_,_1002__)_ = 11.06*** η^2^ = 0.01	*F*_(__1_,_999__)_ = 2.77	*F*_(__1_,_1007__)_ = 0.48

*p < 0.05, **p < 0.01, ***p < 0.001. Comparisons of means (negative and positive perception, unemployment justification and norm) by sex of job-seeker, duration of unemployment, unemployment benefits, and country of the respondents.

Three dimensions of unemployment normalization (negative perceptions, positive perceptions, and justifications) were related to the duration of this period. Thus, the negative perception of unemployment and its external justification tended to increase the longer a period of unemployment persisted. As for positive effects, they were relatively higher at the beginning of this period of career transition, but they faded after about a year of unemployment. For unemployment recurrence, people unemployed for the first time were less likely than others to view it as a normal part of their career and to give it an external justification. People receiving unemployment benefits also tended to give higher rating to the benefits of unemployment. Finally, Luxembourg-based respondents tended to give slightly higher ratings to the perceived negative effects of unemployment than those living in France, as measured by the negative perception factor of the scale. There were no other group differences in the normalization dimensions. Nevertheless, these results must be interpreted with caution because the variance explained in the normalization by each of the demographic variables was less than 4%. Moreover – because the study did not feature longitudinal data about the duration of unemployment – intra-individual variability over time was not analyzed: Only groups of participants unemployed for different lengths of time when they participated in the study were compared with each other.

Finally, correlations were computed between the dimensions of the unemployment normalization and respondents’ age. A significant positive correlation was observed between age and negative perceptions of unemployment (*r* = 0.162, *p* < 0.001). A similar relationship between age and external justifications (*r* = 0.277, *p* < 0.001) was also observed. Thus, older unemployed people tended to have more negative feelings about their situation than younger unemployed people, and they also tended to justify their unemployment situation as being due to circumstances beyond their control, such as social and economic factors. By contrast, age was not related to positive perceptions of unemployment (*r* = −0.048, *p* = 0.128) or the unemployment norm (*r* = −0.032, *p* = 0.321).

### Path Analysis of the Unemployment Normalization

**FIGURE 3:**
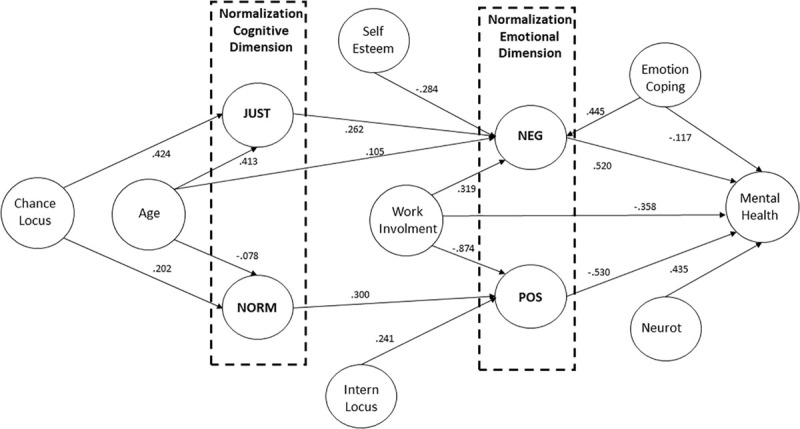
Path analysis of the unemployment normalization. JUST: unemployment justifications; NORM: unemployment norm; NEG: negative perceptions; POS: positive perceptions; Neurot: neuroticism.

In order to verify the impact of all psychological and demographic variables on the unemployment normalization, we ran several analyses. The first was a path analysis including all scales of the study and metric demographic variables. To do this, all items from all psychological scales were introduced into the statistical model to determine the latent psychological variables of the model, and regression analyses were modeled. Finally, a heuristic model of unemployment normalization was computed to provide a better understanding of the influence of individual variables. Figure 3 shows the results of these analyses with satisfactory fit indices (χ^2^ = 15929.89, *df* = 2815, RMSEA = 0.070, CFI = 0.928, TLI = 0.926).

Each latent variable was defined by all the items of the corresponding scale (to simplify, no items were represented in the figure). All the represented paths were significant (*p* < 0.05).

The external justification of unemployment had a positive effect on negative perceptions of unemployment. This same type of relationship was found between the unemployment norm and positive perceptions. These two types of perceptions, positive and negative, were inversely influenced by work centrality. Those who viewed work as important in their lives felt the deleterious effects of unemployment more. The negative effects of unemployment decreased when self-esteem was high and increased with age and emotion-focused coping. The positive effects of unemployment were felt more by those with a more internal locus of control. People who thought that what happened to them was partly due to chance (chance locus) tended to justify their situation more externally and thought a period of unemployment in a career was normal. This trend toward an external justification of unemployment was highly dependent on the age of the unemployed person, and this trend grew over time. Conversely, younger people were more likely to view the period of unemployment as inevitable in life. Finally, we explored the influence of the unemployment normalization on mental health. Those who felt the most negative about being unemployed subjectively felt that they had more problems. The opposite was true for positive perceptions of unemployment. Moreover, perceived mental health also seemed to depend on work centrality, which protected participants from health problems in the same way but to a lesser degree than emotion-centered coping. Finally, neuroticism was positively correlated with the intensity of the problems experienced.

To control for the differential effect of other, non-metric demographic variables in the study, we computed a series of invariance analyses of the previous model. This statistical procedure allows us to confirm if a model is or is not dependent on different groups of people. We used Hirschfeld and von Brachel’s (2014) procedure with the SemTools R-package. The results showed weak invariance for participants’ sex (Δχ^2^ = 42.649, *df* = 65, *p* = 0.985), country (Δχ^2^ = 43.561, *df* = 65, *p* = 0.981), whether they received unemployment benefits (Δχ^2^ = 23.8146, *df* = 65, *p* = 1.000), recurrence of unemployment (Δχ^2^ = 29.534, *df* = 65, *p* = 1.000), and unemployment duration (Δχ^2^ = 56.140, *df* = 195, *p* = 1.000). This indicates that the structural model proposed above did not differ according to these demographic variables when the factor loadings were constrained to be equal. In other words, the proposed model can be considered general enough to represent the process of unemployment normalization for all categories of jobseekers interviewed in this study. It can be considered as being identical for many types of unemployment scenario: men and women; people from both France and Luxembourg; those who do or do not receive welfare benefits; short-term or longer term job-seekers; and people experiencing joblessness for the first-time or not.

Finally, these results fully supported H1 and H2, and partially supported H3 and H4. Thus, the study confirmed the link between work centrality and how unemployment is experienced. Unemployed people for whom work was important in their lives perceived periods of unemployment in a more negative way than others. Conversely, finding some positive aspects to unemployment seemed to be connected to lower feelings of work involvement. These different perceptions were inversely associated with subjective health. In the same vein, unemployed people with more prominent internal causal attribution, did recognize the more positive aspects of unemployment. People with emotion-focused coping mechanisms were more affected by unemployment, felt its negative effects more profoundly, and had lower feelings of well-being. Self-esteem is negatively associated with a negative perception of unemployment, but was not directly linked to well-being. Neuroticism had no impact on negative or position perceptions of unemployment, but there was a direct connection to mental health.

## Discussion

The objective of this study was to better understand the mechanisms of the unemployment normalization first described by Pignault and Houssemand (2017, 2018) in a differential approach. Until now, only a few local socioeconomic variables had revealed that this coping strategy and individual emotional regulation might depend on the social image of unemployment (Houssemand and Pignault, 2017). The negative effects of unemployment should thus be felt more strongly in regions where unemployment is lower, with less history of unemployment, and with shorter periods of labor crises. Conversely, unemployed people in regions more affected by unemployment should have a stronger tendency to find ways to compensate for their job loss, to justify unemployment externally, and to view unemployment as more “normal” within a professional career. These differences were not thought to result from a difference in the negative effects of unemployment between economically different countries but rather to variability in the available emotional regulation and compensation mechanisms. These results clearly emphasize that whereas unemployment always causes significant deleterious effects, the regulation strategies during this period are psychological and individual and can be influenced by the socioeconomic context in which the unemployed live. For example, it may seem more “normal” to experience a period of unemployment in one’s life when a large part of a person’s family, friends, or coworkers have also experienced unemployment. Such regulation and reassessment strategies are possible when unemployment is high locally and is less possible for jobseekers in regions less affected by unemployment. This is therefore not a local and regional habituation to unemployment but an increase in the sources of emotional compensation available individually. In this sense, these results do not contradict research that has described only the deleterious effects of unemployment and considered their constant heavy impact across regions and over decades (e.g., Paul and Moser, 2009). The interest of this new research is to consider the unemployment experience as a more complex process that is based not only on the strong negative effects of unemployment but also on a set of cognitive mechanisms aimed at reducing these effects (Thill et al., 2018). It therefore offers a cognitive approach to analyzing emotional regulation and an understanding of a psychological phenomenon rooted in a socioeconomic context that has potential interactions with the strategies individuals implement.

As such and in order to advance the understanding of these regulatory mechanisms, with this study, we attempted to extend the understanding of how certain psychological constructs and demographic variables influence the unemployment normalization. Drawing on an extensive literature review (Pignault and Houssemand, 2018), a set of psychological variables were selected and linked with the general model of unemployment normalization. Then, a general model of the unemployment normalization and its four dimensions was confirmed as well as their effects on health (Pignault and Houssemand, 2017). The affective variables, *negative* and *positive perceptions* of unemployment, were positively and moderately influenced by the cognitive dimensions of normalization, specifically *external justifications* of unemployment for negative perceptions of unemployment and the *unemployment norm* for positive perceptions. These two cognitive variables depended on the age of the unemployed and their belief in the role of luck or chance in their employment situation (chance locus). Thus, jobseekers who think that chance is responsible for their unemployment attribute their situation more to external factors (companies and the economic downturn) and have a greater tendency to believe that today, unemployment is an inevitable part of one’s career. The age of the unemployed person had a differential effect on the intensity of these two cognitive mechanisms: Younger people believe more that unemployment is a mandatory stage in life, and older people view their employment as dependent on variables they cannot control. The negative effects of unemployment are greater for people with an emotion-focused coping strategy, for those who consider work important in life, and for older jobseekers. On the other hand, there are fewer negative effects for people with high self-esteem. With regard to the positive experiences of unemployment, jobseekers who feel that work is not the only concern in their lives report that being unemployed does not have only disadvantages. This was also the case for those who think that their situation is their responsibility (internal locus). Finally, as expected, negative perceptions of unemployment increased mental health problems and, to the same extent, the opposite was true for positive perceptions of unemployment. Although work centrality mediated the effects of these two dimensions on perceived health, work centrality also had a direct impact on health. It seems to protect against a deterioration in unemployed people’s SWB. The interpretation of this result is rather difficult given the current state of information. It may be the case that the importance of work leads to jobseeking and/or solution strategies that protect these people from health problems. This potential explanation will have to be verified, for example, by introducing questions on the jobseeking activities of the unemployed. In the same vein, emotion-focused coping also had a slight direct effect on mental health by tending to reduce the problems they experienced. Finally, only the personality trait *neuroticism* had a unique direct link to unemployed people’s health, and people with high neuroticism scores tended to report significant health problems.

The main result of these analyses shows that, apart from neuroticism, all the psychological variables are related to mental health only because they influence the dimensions of the unemployment normalization. In fact, the current results confirm most of the conclusions of previous studies but bring to light the need to consider the mechanisms for normalizing unemployment as an intermediate vector of the relationship with jobseekers’ perceived health. Nevertheless, further studies are needed to confirm these results and other variables related to unemployment. A survey on jobseeking techniques should be introduced in order to better understand how the normalization process, beyond the emotional regulation it allows, may influence jobseeking behaviors.

These results are important because they provide a process-oriented understanding of the perception of unemployment, whereas most previous studies, with the exception of Kinicki and Latack (1990) and Latack et al. (1995), only verified correlations between psychological variables and the health of unemployed people. The current results also make it possible to imagine ways for employment and vocational advisors to better address the individual situations of jobseekers. For example, it may be possible to highlight the more positive elements of unemployment in order to reduce the likelihood of experiencing the health problems it produces. More practical studies on new intervention methods would enable us to confirm the present results and the intervention options they propose.

### Limitations

As with any study, certain limitations may mitigate the results that were observed. Although this study offers a cognitive approach and was designed to understand the concept of unemployment normalization and its mechanisms, its focus on multiple dimensions and scales did not allow us to conduct an in-depth exploration of how these effects of compensation may occur between the modes of emotional regulation and how these mechanisms may change over time. Longitudinal studies should be conducted to provide a better understanding of such changes and whether the effects of the psychological variables considered here are constant or change with the duration of unemployment and the jobseeking activities of the unemployed.

The study sample consisted of a relatively limited number of participants, compared to the total population of jobseekers in France and Luxembourg. Moreover, because only volunteers participated in the survey, not every characteristic of the entire population of jobseekers could be taken into account. Even if all unemployed people are required to attend compulsory interviews with the national public employment services, it is conceivable that those who are furthest away from finding work, for objective or subjective reasons, might be poorly represented in this study. Thus, replications of the survey will have to be carried out in order to seek to refine these results and to and to identify differences that may exist between jobseekers.

## Data Availability Statement

The datasets generated for this study are available on request to the corresponding author.

## Ethics Statement

Ethical review and approval was not required for the study on human participants in accordance with the local legislation and institutional requirements. Written informed consent for participation was not required for this study in accordance with the national legislation and the institutional requirements. The Luxembourg Agency for Research Integrity (LARI) specifies that according to Code de la santé publique - Article L1123-7, it appears that France does not require research ethics committee [Les Comités de Protection des Personnes (CPP)] approval if the research is non-biomedical, non-interventional, observational, and does not collect personal health information. Otherwise, with regard to Luxembourg regulations, Code de déontologie médicale, Chapter 5, Article 77 of states “The experimentation on a healthy subject is admitted if it is about a person of major age able to give freely his consent.” Because the present research is not a study for the development of biological or medical knowledge, thus CNER approval is not required.

## Author Contributions

CH was responsible for study conceptualization, data collection, data preparation, data analysis, and report writing. ST was responsible for data collection and data preparation. AP was responsible for study conceptualization, data collection, and report writing. All authors contributed to the article and approved the submitted version.

## Conflict of Interest

The authors declare that the research was conducted in the absence of any commercial or financial relationships that could be construed as a potential conflict of interest.
